# Temporal machine learning for predicting accidents and violations in the mining industry

**DOI:** 10.1007/s40789-026-00943-0

**Published:** 2026-11-09

**Authors:** Nathan Kelley, Pedram Roghanchi

**Affiliations:** Department of Mining Engineering, University of Kentucky, Lexington, KY USA

**Keywords:** Temporal machine learning, MSHA database, Mine accidents, Mine violations, Mining safety

## Abstract

This study investigates the use of temporal machine learning models to predict future accidents and violations at individual mines using historical data from the Mine Safety and Health Administration (MSHA). The primary datasets utilized were MSHA’s Accident, Injuries, and Violations records. Mine accidents were categorized by accident classification, and violations were categorized by relevant Part Sections. The data was cleaned and organized by mine type and commodity, then divided into subsets for training, validation, and testing. A range of configurations, including model type, cutoff thresholds, learning rate, number of hidden layers, data processing methods, data division strategy, observation window length, training subset percentage, and activation functions, were evaluated using the underground coal dataset. These configurations were compared based on F1-score, precision, and recall, identifying the best-performing algorithms. The top-performing models were then tested on the most frequently cited accident types and violation categories across different mine types and commodities. Results showed that the temporal models outperformed random chance, particularly for high-frequency incident types, indicating that such models can aid in anticipating future safety events. The underground coal dataset yielded the highest F1-scores, suggesting that model performance may vary depending on dataset characteristics and selection criteria. Predictive performance further improved when combining top-performing F1-score algorithms with those optimized for precision or recall, or by adjusting prediction cutoff thresholds.

## Introduction

**Fig. 1:**
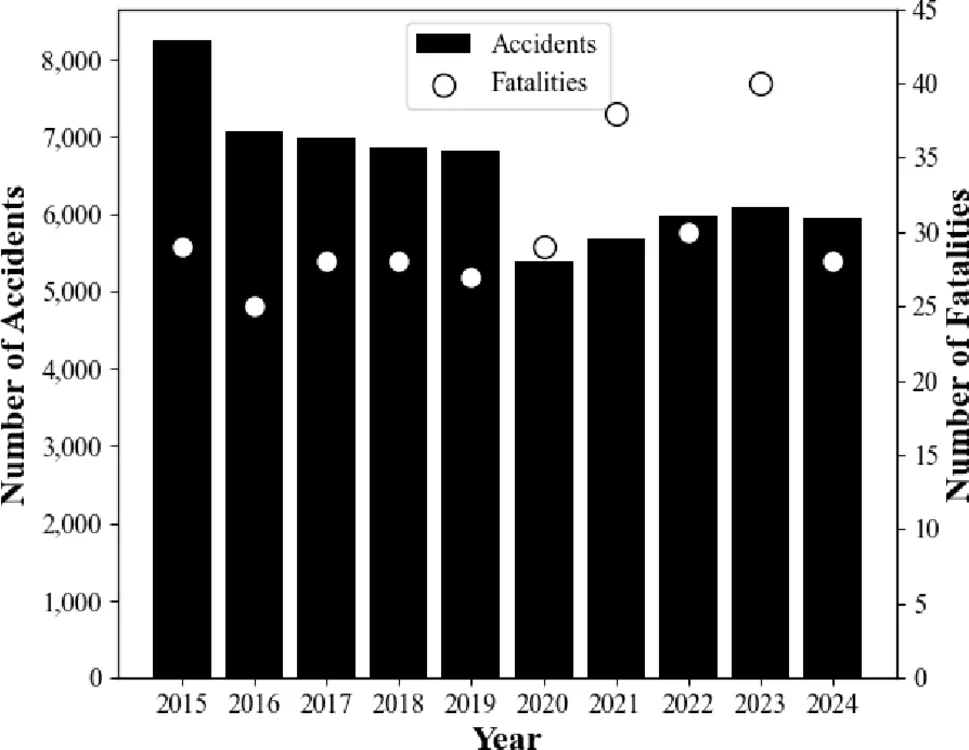
Number of recorded accidents and fatalities in the mining industry in past decade (MSHA 2025a). The data indicates that while the number of reportable accidents has been decreasing, the number of fatalities has been increasing

The mining industry is characterized by a high-risk work environment with operational, geological, mechanical, and environmental factors posing hazards to workers. Figure 1 breaks down the reported accidents and fatalities in the mining industry over the last decade. The data shows that although reportable accidents have been declining by about 230 incidents annually, the number of fatalities has been rising by about one each year (MSHA 2025a). Technological innovations have enhanced workforce safety in the mining industry. An example of this advancement was the introduction of the first continuous miner unit in the United States in 1948 (Ramani 2025). In the years that followed, technological advancements continued to enhance the safety of miners with innovations such as mechanical roof bolters, four-gas monitors, and programmable blasting systems (MSHA 2025c). A distinct subsect of these technologies, classified as automation, has been used to keep miners away from hazardous conditions and increase efficiency since the 1960s and 1970s (McNab and Garcia-Vasquez 2011). Automation is defined as a task performed by a machine that was once performed by a human (Parasuraman and Riley 1997).

Initially, the primary uses for these autonomous systems were to perform repeated tasks that required speed and precision humans were incapable of (Long et al. 2024). Through the implementation of machine learning (ML), these systems have been able to be utilized for more complex and demanding functions. An example of this implementation is in self-operating machinery. As powered haulage and machinery consistently accounting for the two most common fatal accident classifications, significant effort has been directed towards their automation to reduce human exposure to hazardous situations and support operators during production (MSHA 2025c), including applications such as autonomous haul trucks (Voronov et al. 2020) and excavators (Ramezani and Tafazoli 2020).

Machine learning in mining has functions beyond robotics as it is also prevalent in the field of data analytics. Every day, mines make predictions based on collected data that affect the outcome of workers’ health and safety. Large and complex datasets pose challenges for interpretation through manual analytical methods, thereby motivating the application of machine learning techniques as a means of extracting meaningful patterns, trends, and insights from the datasets (Mahesh 2020). Through this analysis of data, a predictive model can be developed to predict future data based on identified trends and patterns in previous datasets. Many researchers have focused on ground control issues due to the availability of their data and severity in the event of failure. This includes roof falls (Anani et al. 2023), roof displacements (Li et al. 2025), pillar stability (Quevedo et al. 2024), and rock bursts (Shukla et al. 2021, Shao and Chen 2023, Miao et al. 2024). The models utilize datasets already available to mines, such as pillar dimensions, rock properties, geological conditions, and production schedules, allowing them to train their models readily, and resulted in reasonable success among their models. Models have also been trained to predict mine emergencies. Using factors such as borehole data, water source, and water channel, models were developed to predict water inrush (Yin et al. 2023) or improve upon existing water inrush models (Wang et al. 2020). Similarly, using previous underground coal mine fire data, researchers have developed models to predict fire size and response time (Barros-Daza et al. 2022) and, by utilizing fire-related parameters, predict fire intensities (Kamran and Shahani 2022). Although mines readily collect the data used in many of these models, consolidating and gaining access to these datasets from these mines can be difficult and time-consuming.

The complex nature of the Mine Safety and Health Administration’s (MSHA’s) datasets, along with their availability, makes it ideal for machine learning applications. Multiple researchers have used aspects of these datasets to predict health and safety outcomes. Using the accident dataset, models were created to identify the factors that lead to lost time events (Kahraman 2021), predict the degree of future injuries (Manjunatha 2023), predict the outcome of events (Yedla et al. 2020), and determine the risk of future fatalities (Kinilakodi and Grayson 2012). Using the violation dataset, researchers employed machine learning to identify trends between the reported violations and the subsequent accidents (Gernand 2014). This highlights the research potential of machine learning in mining datasets, as multiple researchers use different models and data processing methods to create models that frequently achieve success.

We investigate to answer the following research question: can temporal deep learning models be used to predict the occurrence of specific types of accidents or violations at a given mine, based on its history? In this study, we develop temporal deep learning models (using LSTM/GRU architectures) to predict specific categories of mining accidents and violations at the mine level. Though many different models are used in previous literature, the use of temporal models is uncommon. This means that traditionally, datapoints are looked at as isolated occurrences for prediction. We argue that although an individual datapoint may be significant, specific sequences of datapoints can also indicate the occurrence of a future incident, enabling more accurate predictions without the need to expand the dataset. This study looks to take the LSTM/GRU architectures and tune their individual hyperparameters to optimize it for predicting accidents and violations in a mining application. Furthermore, predictive models for predicting specific violations or accidents are uncommon. By focusing on specific accidents and violations we increase the difficulty of the task by dividing our dataset more but provide applicable results for not just fellow researchers but to the mines themselves. Based on the predictions, a mine can retrain and thus prepare workers for specific accidents and violations before they are likely to occur. This represents a novel application of deep learning to mine safety data, expanding on prior works that did not account for the order of occurrences and by outputting a result relevant to mine operators for future training opportunities.

## Materials

### MSHA datasets

The three datasets used in this study were the *Accident Injuries*,* Violations Dataset*, and the *Mine Address of Records* dataset. As of June 3rd, 2025, the accident dataset contained 267,455 entries and the violation dataset contained 2,996,553 entries. Each entry in the accident dataset was defined by 57 attributes and each violation had 61 attributes (MSHA 2025a). These attributes include characteristics of the entries such as the mine where the incident occurred, when it occurred, and the subsequent fine.

According to MSHA, a mine accident is an event at a mine that results in death or injury, or that has the potential to cause death or injury (Administration 1977). The accident-injury dataset is comprised of all accidents, injuries and illness reported to MSHA in form 7000-1 (MSHA 2025a). This form must be submitted within ten days and failure to do so will result in punishment under 30 U.S.C § 818 and 30 U.S.C § 820(a) (Administration and Labor 2024). As defined by MSHA, an occupational injury includes those cases which result from a work accident or from an exposure involving a single instantaneous incident in the work environment such as twisting an ankle. If an injury occurs from a prolonged exposure, it would be classified as an illness like a sunburn (Administration and Labor 1986). The violations dataset is comprised of all violations issued at mines in MSHA’s jurisdiction. Citations, or violations, are issued if upon inspection, a mine is in violation of the Federal Mine Safety Act of 1977 or a mandatory health or safety standard, rule, order, or regulation related to the act (Administration and Labor 2020), MSHA will issue a citation which can come with a monetary fine or a withdrawal from operations (Congress 1977). These are issued during mine inspections that take place at least once a quarter for underground mines or twice a year for surface mines. More inspections could be made if the mine contains high levels of toxic or explosive gases, to check for compliance of hazardous conditions, or if a fatality/injury occurred (Congress 1977). For this paper “accident” refers to any entry in the accident injury dataset, “violation” refers to any entry in the violations dataset, and “incident” refers to either.

### Temporal machine learning algorithms

For this study, a machine learning classifier algorithm was employed. A classifier uses a series of inputs to produce a single result (Domingos 2012). It achieves this by assigning weights to the various inputs to determine the extent to which each should influence the output. These weights are determined through the learning process of the machine learning model (Mitchell 1997). This learning process is conducted by using the algorithm when the outputs are already known and adjusting the weights accordingly (Russell and Norvig 1995). The inputs are multiplied by their respective weights, summed, and pass through an activation function. If the outputs are then used as the inputs for another machine learning algorithm, this first set of outputs is classified as a hidden layer. The entire framework is called a neural network (Russell and Norvig 1995). Neural networks can vary significantly depending on the hyperparameters selected. Hyperparameters must be determined before training and are used to configure aspects of the algorithm resulting in vastly different performance results (Claesen and Moor 2015). Some hyperparameters altered in this study are as follows:

*Activation functions* Of the possible activation functions the ones utilized in this study include sigmoid, hyperbolic tangent (tanh), basic rectified linear unit (ReLU), and leaky rectified linear unit (leaky ReLU). The formulas and output ranges for each activation function are outlined in Table 1.

**Table 1:** Activation functions utilized in this study (Popescu et al. 2009, Szandała 2020)

Activation function	Formula	Output range
Sigmoid	$\:\frac{1}{1+{e}^{-x}}$	(0,1)
Tanh	$\:\frac{{{e}^{x}-e}^{-x}}{{e}^{x}+{e}^{-x}}$	(-1,1)
ReLU	$$\:\left\{\begin{array}{c}0\:if\:x<0\\\:x\:if\:x>0\end{array}\right.$$	[0,∞)
Leaky ReLU	$$\:\left\{\begin{array}{c}x\:if\:x\le\:0\\\:ax\:if\:x>0\end{array}\right.$$ (in this study *a* = 0.03)	(-∞,∞)

**Fig. 2:**
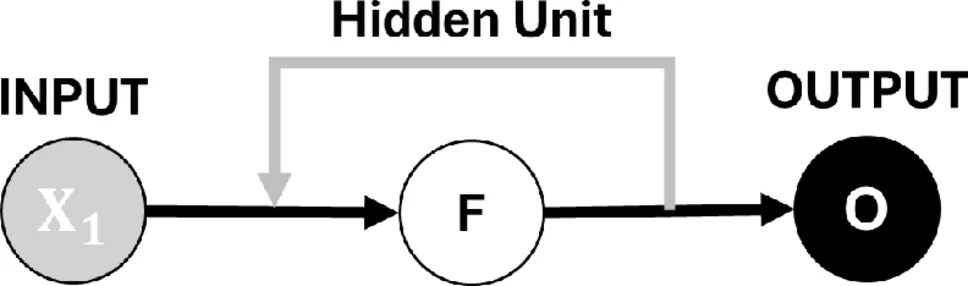
Recurrent neural network diagram (Goodfellow and Courville 2016, Rumelhart et al. 1986)

*Model* This study looked to focus on a temporal machine learning model, normally referred to as “architecture”. This meant that not only the individual data point features, but also the order of occurrences, would influence the prediction model. For this reason, two different types of recurrent neural networks (RNNs) were investigated being Long-Short Term Memory (LSTM) and Gated Recurrent Units (GRUs). RNNs allow for a sequence of data to be inputted instead of inputs individually by using a hidden state to carry information over between inputs (Rumelhart et al. 1986) shown in Fig. 2.

**Fig. 3:**
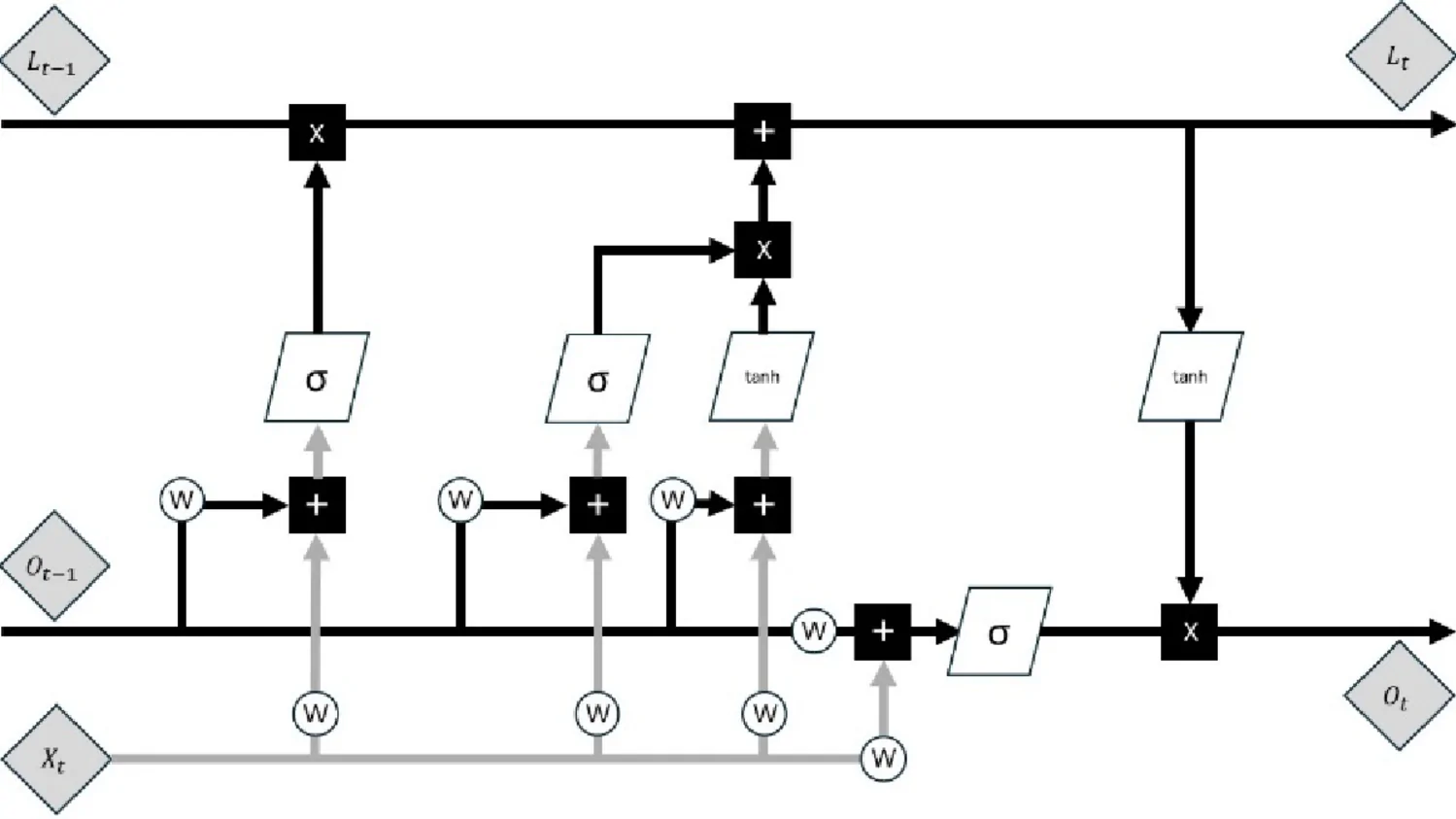
LSTM diagram with *O* as the output, *l* as the long-term memory, *X* as the input, *t* as the number in the sequence, *W* as the weights of their respective branches, *σ* as a sigmoid activation function, and tanh as a tanh activation function (Hochreiter and Schmidhuber 1997)

Both LSTMs and GRUs looked to improve on RNNs by using a series of gates to combat the vanishing and exploding gradient problem prevalent in traditional RNNs (Hochreiter and Schmidhuber 1997). A diagram of an LSTM is shown in Fig. 3 with O as the output, L as the long-term memory, X as the input, t as the number in the sequence it is in, W as the weights of their respective branches, σ as a sigmoid activation function, and tanh as a tanh activation function. In Eqs. (1)–(6) (Dey and Salem 2017), W and U represent weights, and b denotes the bias term; subscripts identify the corresponding parameter. The functions *σ* () or tanh () represent going through a sigmoid or tanh activation function respectively. An LSTM is comprised of three gates and two strings of memory. The top line represents the long-term memory and the second to last bottom line represent the short-term memory which both begin at zero. With each input, the long-term memory is updated and the short-term memory becomes the previous iteration’s output. The first gate is represented by the leftmost arrow connected to the long-term memory and Eq. (1) (Dey and Salem 2017). The short-term memory and current input are summed after being multiplied by their respective weights and multiplied by the previous long-term memory. This is called the forget gate because it determines how much of the previous long-term memory is carried forward. The second gate, the input gate, is represented by the middle-most arrow connected to the long-term memory and Eq. (2) (Dey and Salem 2017). The short-term memory and current input are multiplied by their respective weights and summed. This process is repeated twice with one output going through a sigmoid activation function and the second going through a tanh activation function. These values are multiplied together and added to the long-term memory. The final gate, the output gate, is the final vertical arrow on the long-term memory and shown in Eq. (3) (Dey and Salem 2017). The short-term memory and current input are multiplied by their respective weights, summed, passed through a sigmoid activation function, and multiplied by the long-term memory after it had been passed through a tanh activation function. This value is the output and the next short-term memory (Hochreiter and Schmidhuber 1997).

1 $$\:F={L}_{t-1}*\sigma\:({W}_{F}{X}_{t}+{U}_{F}{O}_{t-1}+{b}_{F}$$

2 $$\:{L}_{t}=Z+\mathrm{t}\mathrm{a}\mathrm{n}\mathrm{h}\left(B*\sigma\:\right({W}_{I}{X}_{t}+{U}_{I}{O}_{t-1}+{b}_{I}$$

3 $$\:{O}_{t}=\sigma\:\left({W}_{R}{X}_{t}+{U}_{R}{O}_{t-1}+{b}_{R}\right)*\mathrm{t}\mathrm{a}\mathrm{n}\mathrm{h}\left({L}_{t}\right)$$

**Fig. 4:**
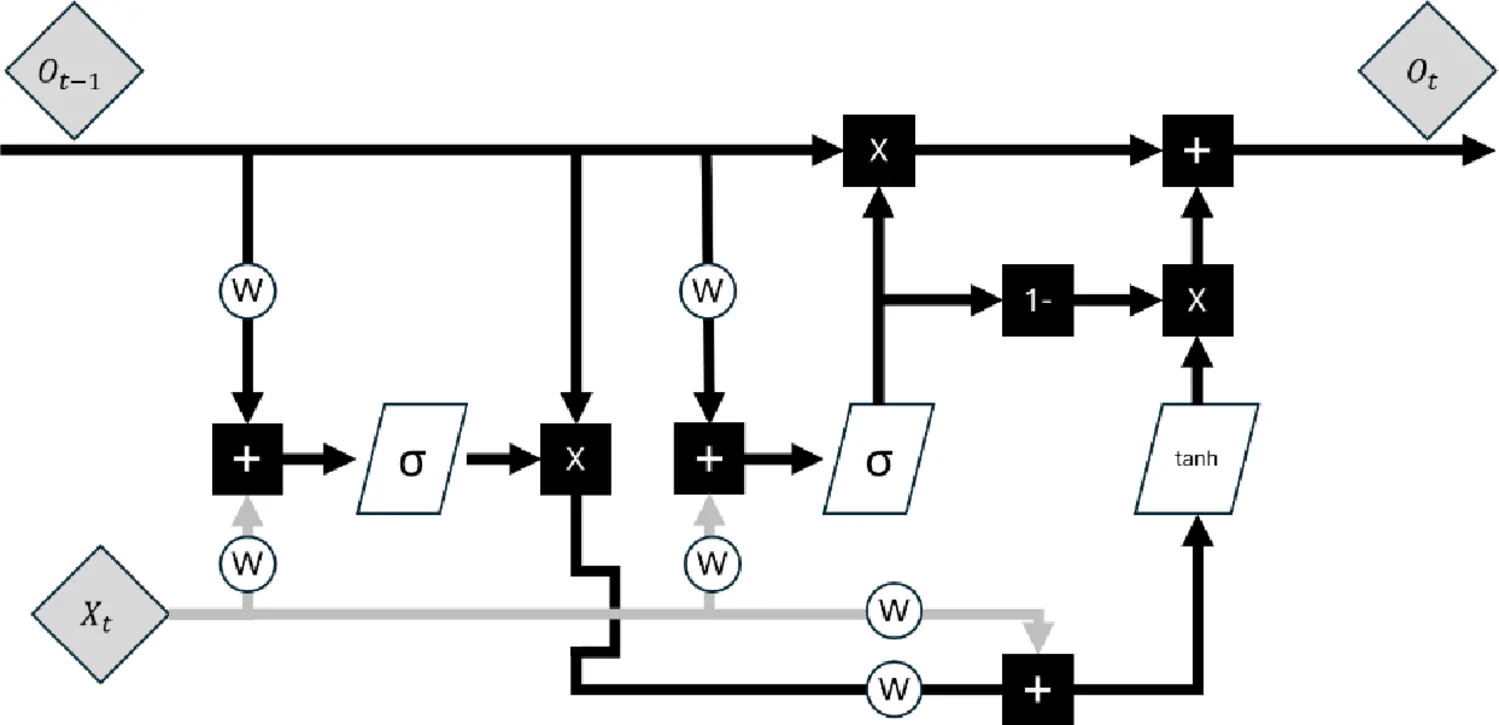
GRU diagram with o as the output, *x* as the input, *t* as the number in the sequence, *W* as the weights of their respective branches, *σ* as a sigmoid activation function, and tanh as a tanh activation function (Cho et al. 2014)

The GRU is a simpler system with only an update gate ($\:{z}_{t})$ and a reset gate ($\:{r}_{t})$ represented by Eqs. (4) and (5) (Cho et al. 2014). The output for a GRU is generated by Eq. (6) (Cho et al. 2014). Gru is visually represented in Fig. 4.

4 $${z_t} = \sigma ({W_z}{X_t} + {U_z}{O_{t - 1}} + {b_z})$$

5 $${r_t} = \sigma ({W_r}{X_t} + {U_r}{O_{t - 1}} + {b_r})$$

6 $${O_t} = \left( {1 - {z_t}} \right)*{O_{t - 1}} + {z_t}*{\rm{tanh}}({W_h}{X_t} + {U_h}\left( {{r_t}*{O_{t - 1}}} \right) + {b_h})$$

## Methods

### Data processing

The datasets are downloaded as text files and are subsequently inoperable. A Python based application was used to allow for implementation into the machine learning algorithms primarily using the Pandas library (McKinney 2010).

A single attribute was chosen from each dataset to be the prediction attribute. This attribute had to meet two criteria: Have a limited number of unique entries and be able to extract meaningful information from the predictions. In 2024 an accident occurred every 0.00001 man-hours worked (MSHA 2025a). If the datapoints are divided into number of occurrences per month, the vast majority of points will have no incidents. This number further decreases due to our desire to predict not just an accident will occur but a specific accident. This data imbalance will lead to the program greatly favoring “no incident” (Blagus and Lusa 2013) based more on frequency then trends. Second, is the need for the predicted point to convey meaningful information to the mine operator. For accidents, the simplest prediction would be to predict an accident would occur but without any further information this prediction is too broad for a mine operator to take steps to prevent its occurrence. The goal is to generate a prediction in which the mine operator can take meaningful steps in the form of alerting or retraining miners to prevent it from occurring. The inspiration behind this idea was MSHA’s fatality alerts. These are one-page documents outlining how the fatality occurred, and what steps can be taken to prevent it from reoccurring (MSHA 2025c). For the accident dataset the chosen attribute was “CLASSIFICATION” which identifies the circumstances which most contributed to the accident. For the violation dataset the “PART_SECTION” attribute was chosen due to it identifying what part or section of Title 30 CFR was violated. If the entry had a subheading, it was dropped to limit entries while still maintaining relevancy.

Each dataset was converted into a data frame. To begin with all data prior to 2010 was removed from the datasets. Due to the constant need to improve safety, new regulations and laws are added annually to the mining industry (MSHA 2025b). Coincidentally, the factors leading to various accidents and violations are altered by this changing legislation. Therefore, data points prior to 2010 were excluded to ensure a sufficiently large dataset for training the predictive model while maintaining consistency in the factors contributing to the events. The violation dataset was reduced to the following attributes: *INSPECTION_BEGIN_DT*, identify date of violation; *MINE_ID*, identify mine where violation occurred; *MINE_NAME*, was not inevitably used for this study; and *PART_SECTION*, main attribute to be predicted (MSHA 2025a). The accident dataset was reduced to the following attributes: *MINE_ID*, identify mine where accident occurred; *ACCIDENT_DT*, identify date of accident; *CLASSIFICATION*, main attribute to be predicted; *CLASSIFICATION_CD*, code of main attribute to be predicted; *DEGREE_INJURY*, used as an additional datapoint, *ACCIDENT_TYPE*, was not inevitably used for this study; and *ACCIDENT_TYPE_CD*, was not inevitably used for this study (MSHA 2025a). This reduction was due to the majority of attributes being redundant or not applicable to the purposes of the program. This decreased storage requirements and improved processing speed by removing non-necessary attributes from the datasets. The *MINE_NAME*, *ACCIDENT_TYPE*, and *ACCIDENT_TYPE_CD* originally planned to be used over other chosen values and were never removed but did not affect the final program. The *CLASSIFICATION* attribute was entangled with the *CLASSIFICATION_CD* attribute. Additional processing was developed to sort and divide these two attributes into their appropriate columns. The *DEGREE_INJURY* attribute was deemed relevant but distinct from classification, allowing it to be used as an additional datapoint in the machine learning process. The accident and violation datasets were combined so that that the model could learn cross-effects between violations and accidents to identify the trends of what sequence of incidents leads to future incidents.

A blank array was created for each mine ID with each row represented a unique violation part section, accident classification, or *DEGREE_INJURY* and each column represented a quarter of a year from 2010 to 2024. The time periods we set at quarters due to the sparce nature of accidents and certain violations. This temporal compression changes the impact for an annual incident from 8% per year to 25% per year while still making the possible prediction relevant. The entire array was populated with zeros. The program then iterated through each entry in the combined dataset added one to the appropriate mine ID sheet based on the type of incident and the quarter on which the incident occurred. Each unique accident, violation, and degree of injury was normalized with every entry in its row across mine IDs.

The data had to be divided into input and output data. For each mine, we constructed sequences of length N (e.g., when *N* = 5 corresponds to five quarters) that encapsulated all types of accidents, violations, and degrees of injury that occurred in those consecutive N quarters for the input data. The cell directly after the input data of the incident for that chosen dataset was labeled as the output data. Table two illustrates this process on a simulated mine and displaying only two rows of accidents, violations, and degree of injury (DOI). In this example, *N* = 5, and the selected incident is “Accident #1”, with input data highlighted in grey and output data highlighted in black. We then slide this window by one quarter and repeat, thereby generating multiple training samples per mine. Each incident had its own unique datasets (Table 2).

**Table 2:** Example limited mine array (*N* = 5)

Year	2010				…	2023				2024			
Quarter	1	2	3	4	…	1	2	3	4	1	2	3	4
Accident #1	0	0	0	1	…	0	1	**0**	**0**	**1**	**1**	**1**	***0***
Accident #2	0	0	2	0	…	0	1	**1**	**0**	**0**	**0**	**0**	1
Violation #1	0	3	1	1	…	0	1	**1**	**1**	**0**	**1**	**0**	0
Violation #2	2	1	2	1	…	0	1	**1**	**0**	**1**	**0**	**0**	0
DOI #1	0	0	0	0	…	0	1	**1**	**0**	**0**	**0**	**0**	1
DOI #2	0	0	0	1	…	0	1	**1**	**0**	**1**	**1**	**1**	0

Selected Incident: Accident #1; Input Data in bold; Output Data in bolditalics

Each output data point is converted into a binary value, 1 if the incident occurred and 0 if it did not. For the purposes of this experiment, the only concern was the incident’s occurrence; thus, by converting into binary values it eliminated unnecessary variability. The data was divided into training and testing datasets with 90% allocated to training and the most recent 10% was reserved for testing. The most recent 10% of the data is used for testing because it represents the closest complete dataset to the future data the final program aimed to predict. Then 10% of the training dataset was sectioned off for the validating dataset.

To further enhance the accuracy of the program the data was divided by mine type-surface, underground, and facility- and by mined commodity-coal, metal, and nonmetal due to the violations, accidents, and overall influencing factors varying between these groups. This allows the user to only use models trained on a specific mine’s type and commodity. From the Mines Addresses of Record dataset, the type of each mine ID was determined from the *MINE_TYPE* attribute. The mined commodity for each mine ID was determined first by the *COAL_METAL_IND* which showed if a mine mined coal or metal/nonmetal. Metal and nonmetal IDs were separated by the *PRIMARY_SIC_CD* attribute. This attributed indicated the specific commodity being mined; if the mined commodity contained a term associated with metal commodities (e.g., copper, gold), it was classified as metal, otherwise it was classified as nonmetal.

As stated previously, the data is highly imbalanced. After the consolidations of the data into quarters the third most cited violation and fourth most frequent accident only occurred in between 26% and 36% and 3–9% of the underground coal datapoints. To combat this further, we employed oversampling and combined over/under-sampling techniques via the imbalanced-learn (imblearn) library. Specifically, we generated training sets with class ratios of 50:50, 60:40, or 70:30 (positive: negative), using three methods: synthetic minority oversampling technique (SMOTE), a combination of SMOTE and Tomek links (SMOTETomek), or adaptive synthetic sampling (ADASYN) (LemaÃŽtre et al. 2017). SMOTE is an oversampling techniques that generates additional datapoints by interpolating between the characteristics of existing datapoints (Blagus and Lusa 2013). SMOTETomek takes the process of SMOTE but also applies the under-sampling method of Tomek links. Tomek Links identifies and removes points in the majority class with similar parameters as the minority class to increase the clarity in the divide between classes (Viadinugroho 2021). Like SMOTE, ADASYN is an oversampling method that generates new datapoints between existing datapoints. Unlike SMOTE, the number of points generated between two points in ADASYN is based on its number of nearest neighbors (K) that are in the majority class (M_*i*_) to calculate a points weight (W_*i*_) shown in Eq. (7) (He et al. 2008). This allows for more points to be generated around minority class points that are surrounded by majority class points (He et al. 2008). These methods have been employed to improve the quality of various imbalanced datasets including crime (Angondji et al. 2024), construction accidents (Koc et al. 2022), hypothyroid disease (Prantik et al. 2024), and the MSHA datasets (Manjunatha 2023) for machine learning applications. The data transformation was only applied to the training data to retain the maximum possible integrity for the testing data.

7 $$\:{W}_{i}={M}_{i}/K$$

### Machine learning algorithms structure

Each algorithm used the keras library in python (Chollet 2015) with an adaptive moment estimation (ADAM) optimizer with a binary cross-entropy loss function. The equation for the loss function is shown in Eq. (8) with $\:{t}_{n}$ representing the true value and $\:{y}_{n}$ representing the predicted value (Wicaksana and Rachman 2018). The formulas for parameter updating for ADAM with * t* being the current iteration, P as the parameters, g the gradient of the loss function, and lr as the learning rate can be seen in Eqs. (9)-(11) (Kingma 2014). Each algorithm ended in a sigmoid activation function which resulted in a value ranging from 0 to 1. If the resulting value was 0.5 or higher, the incident was predicted to occur.

8 $$\:E\left(w\right)=-\mathrm{ln}\mathrm{p}\left(t|w\right)=-\sum_{n=1}^{N}\{{t}_{n}\mathrm{ln}\left({y}_{n}\right)+\left(1-{t}_{n}\right)\mathrm{l}\mathrm{n}(1-{y}_{n})$$

9 $$\:{M}_{t}=0.9*{\stackrel{-}{M}}_{t-1}+(1-0.9){g}_{t}$$

10 $$\:{v}_{t}=0.999*{v}_{t-1}+(1-0.999){{g}_{t}}^{2}$$

11 $$\:{P}_{t+1}={P}_{t}-lr*\frac{\frac{{m}_{t}}{1-{0.9}^{t}}}{\sqrt{\frac{{V}_{t}}{1-{0.999}^{t}}+{10}^{-7}}}$$

**Fig. 5:**
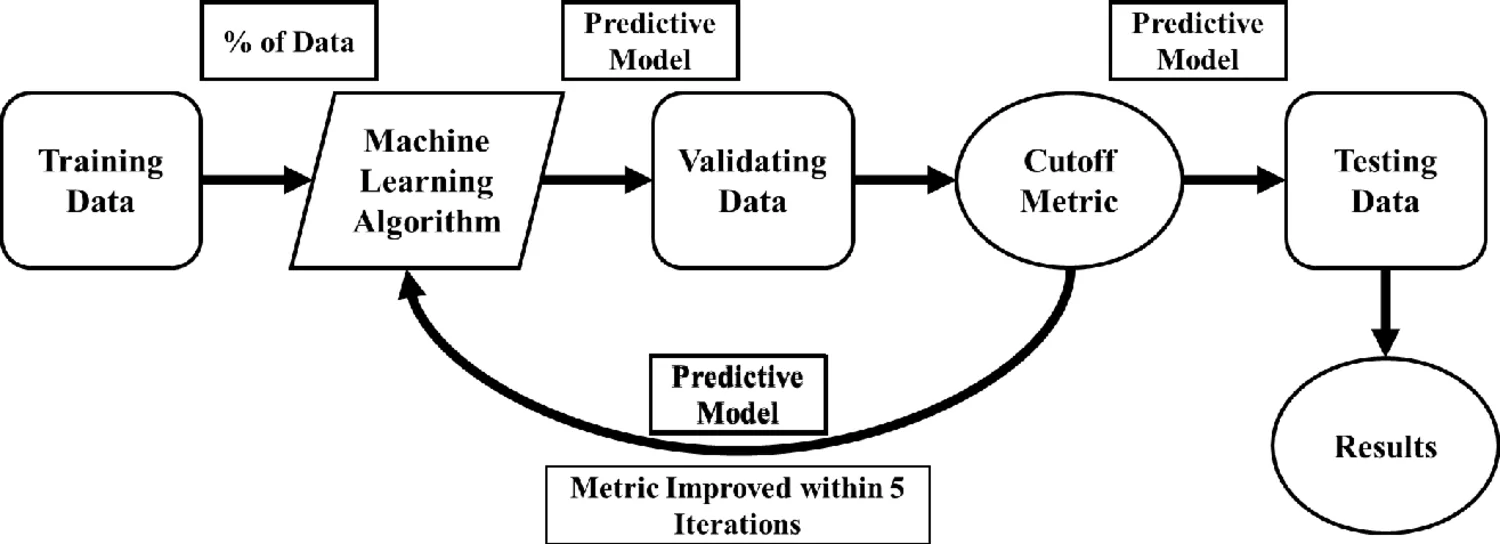
Machine learning algorithm testing flowchart

Each machine learning algorithm followed the same procedure during testing as shown in Fig. 5. A percentage of the chosen training data is introduced into the machine learning algorithm to identify the trends in the dataset. By only inserting a percentage, this decreases the likelihood the subsequent model will be overfitted to the training data and decreases run-time. Due to the algorithm being presented with both the inputs and outputs of the data, the algorithm can determine what accidents, violations, degrees of injuries, or the sequences of them lead to the chosen incident occurring. This is so that when the outputs are not presented, the model can you these trends to make predictions on what the outcome could be. The weights calculated by the algorithm for each input are then expressed as the predictive model. Only inputs of the validating dataset are then passed through the predictive model. The predictive model then outputs a prediction for each input. The cutoff metric score is then calculated based on how closely the model’s predictions matched the know outputs for the provided inputs. A single iteration is now complete, and another percentage of training data is introduced to the machine learning algorithm along with the previously introduced training data. This cycle will repeat until the given cutoff metric does not improve within five iterations at which point the most optimal predictive model is tested on the testing dataset. This process is identical to the process used in the validation dataset, but a different score was calculated separate from the cutoff metric. Each model only predicts one unique incident due to each dataset only contains outputs from one unique incident.

The four possible outcomes of predictions are presented in the confusion matrix in Table 3 (Kohavi and Provost 1998): true positive (TP) - an incident was predicted and an incident occurred; true negative (TN) - no incident was predicted and no incident occurred; false positive (FP) - an incident was predicted and no incident occurred; false negative (FN) - no incident was predicted an incident occurred. The primary goal of this study was minimizing the number of false negatives displayed by the models. For accidents, a false positive could result in a time loss, whereas a false negative could result in severe injury. Although less critical, false positives still play a vital role in increasing the perceived reliability and trustworthiness of the program. By reporting an incident as occurring 100% of the time, there would, in turn, be no false negatives, thus meeting the presented expectations. This program would not be used, however, due to it providing unreliable information. A balance between the two is vital. This thought led to the choosing of the primary grading metric for analyzing the quality of the predictions.

**Table 3:** Confusion matrix (Kohavi and Provost 1998)

Item		Predicted	
		TRUE	FALSE
Actual	TRUE	True Positive (TP)	False Negative (FN)
	FALSE	False Positive (FP)	True Negative (TN)

Upon initial inspection, accuracy was to be the primary grading criterion with the equation for accuracy being shown below in Eq. (12) (Kohavi and Provost 1998). Due to retaining the data imbalance in the testing dataset, accuracy would severely misrepresent the quality of the model. If a particular dataset had 98% negative points, then a model that always reports “No Incident” would be 98% accurate, deeming the model a success. This approach maximizes the number of false negatives which would directly oppose the main project goal. Recall was then considered, as shown below in Eq. (13) (Rijsbergen 1979), which solely prioritizes minimizing false negatives. By neglecting to include false positive points, this leads to models that do encapsulate most positive points but are untrustworthy due to high volumes of false alarms. Conversely, precision, defined in Eq. (14) (Rijsbergen 1979), is very trustworthy in its positive predictions but by doing so fails to alert the user of multiple positive values. F1-Score considers both false negative and false positive values without becoming overcome by the large number of true negative values by combining recall and precision shown in Eq. (15) (Rijsbergen 1979). The F1-score is a common performance metric in medical image segmentation, as it prioritizes true positives and omits true negatives (Müller et al. 2022), which is analogous to our focus here. F1-Score was used as the grading metric for the final results of each model to determine quality.

12 $$\:Accuracy=\frac{TP+TN}{TP+TN+FP+FN}$$

13 $$\:Recall=\frac{TP}{TP+FN}$$

14 $$\:Precision=\frac{TP}{TP+FP}$$

15 $$\:F1-Score=\frac{2*Precision*Recall}{Precision+Recall}$$

### Machine learning algorithm testing

**Fig. 6:**
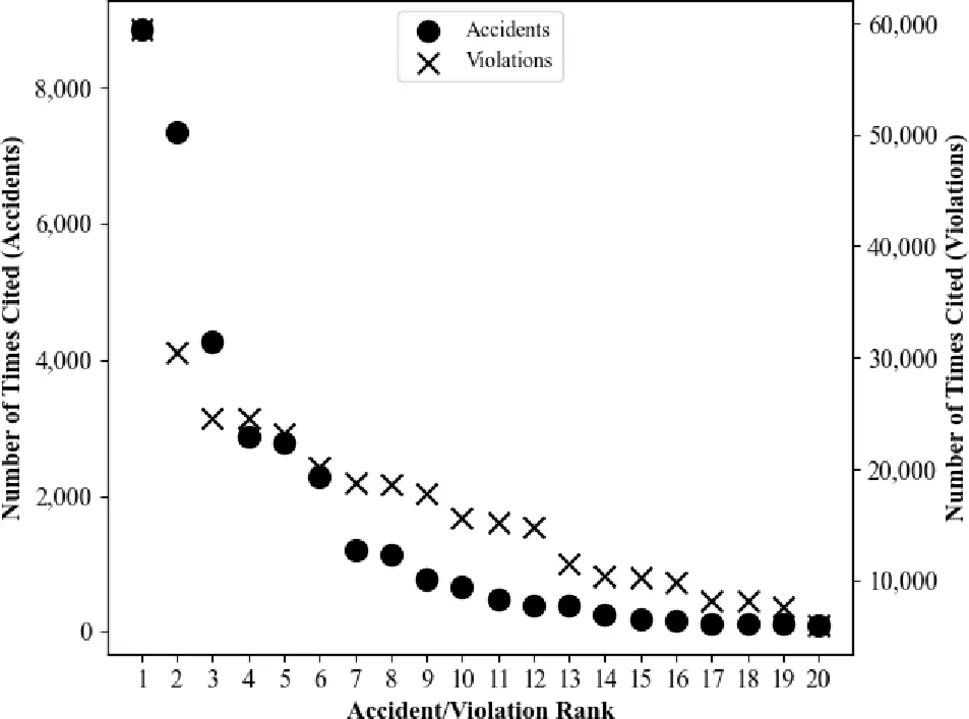
Twenty most cited accidents (circles) and violations (crosses) for underground coal mines in the us from 2010–2024 with data from November 2024 (MSHA 2025a).

Testing the algorithms was broken up into three distinct stages being the initial, primary, and final testing stages. The initial and primary testing stages were defined by determining the optimal algorithm based on the underground coal dataset. The underground coal dataset was chosen due to the assumption that it would be the most balanced in terms of naturally positive (incident occurred) and negative points (incident did not occur). Final testing tested the most optimal algorithms on datasets the algorithms were not chosen from. We selected the fourth most frequent accident category (Machinery, in underground coal) and the third most frequent violation code (30 CFR § 75.1731) as the prediction targets for initial experiments because the top one or two categories had disproportionately high occurrence counts. By choosing a slightly less frequent incident, we avoided extreme class imbalance in the target variable while still focusing on a point more representative of the population. Figure 6 illustrates that the most cited category far exceeds the average, hence our use of the 3rd/4th ranked categories as representative targets. All statistical analysis was conducted using *Minitab Statistical Software* (Minitab 2025).

For the initial testing stage, the following hyperparameters remained constant throughout testing unless they were the hyperparameters being tested at that time:

- Model: GRU.
- Learning Rate: 0.001.
- Number of Observed Points: 5.
- Percentage of Training Data per Iteration: 2%.
- Cutoff metric: AUC.

Each hyperparameter being tested was tested 360 times with ten tests for each combination of activation functions (Leaky ReLU, ReLU, or Tanh), data processing types (SMOTETomek, SMOTE, ADASYN, or none), and data processing divisions (50/50, 60/40, or 70/30) to observe multiple scenarios for each tested hyperparameter. The hyperparameters being tested were the cutoff metric, AUC or F1-Score; model, GRU or LSTM; learning rate, 0.01,0.001, or 0.0001; and the number of hidden layers, 1,2,3,4, or 5. To determine if the results were statistically significant, first a blocking design was employed to control variability through the varied hyperparameters. Following ANOVA, pairwise comparisons were conducted using both Tukey’s HSD and Fisher’s LSD tests at a 95% confidence level (α = 0.05). The best performing hyperparameter options based on F1-score we carried into the primary testing stage.

In the primary testing phase the activation functions (Leaky ReLU, ReLU, or Tanh), data processing types (SMOTETomek, SMOTE, ADASYN, or none), data processing divisions (50/50, 60/40, or 70/30), number of points observers (5, 10, 15, or 20), and the percentage of training data per iteration (6.67%, 4%, 2%, or 1%) were varied in each dataset with each algorithm being tested ten times. The mean F1-Score, precision, and recall values were calculated for each hyperparameter combination and the best performing algorithms for each metric and dataset were carried into the final testing phase. To investigate if each variable being tested had a significant effect on the F1-score, a general factorial regression with a 95% confidence level was conducted on both the accident and violation results.

Phase one of the final testing phase consisted of the best performing F1-Score algorithms for the accident and violation datasets being tested on multiple different accidents and violations in the underground coal datasets. The objective of phase one was to view the algorithms performance on predicting other accidents and violations while remaining in the underground coal dataset. Accidents were tested 70 times and violations were tested at least 20 times. The F1-scores were graphed against the number of occurrences. A trendline was displayed for each dataset and the coefficient of determination was used to determine how well the trendline fit the data. The coefficient of determination, R^2^ value, quantifies the extent to which the current regression model represents the relationship between the dependent and independent variables (Nagelkerke 1991). This value ranges from one, meaning the model fits the data perfectly, to zero, meaning the model does not fit the data at all. Phase two of the final testing phase consisted of testing the top performing F1-Score algorithms again but this time on the 10 most cited accidents and violations in the surface and underground coal, metal, and nonmetal datasets. The surface nonmetal database dataset was restricted to only include points from 2021 to 2024 for the violations and 2020–2024 for accidents. This was due to the size of the surface nonmetal database being too great for the random-access memory (RAM) of the systems being used. Accidents and violations were tested at least 20 times. The F1-scores were first graphed against the number of occurrences per mine per year to standardize the data across datasets. Now the algorithms will be tested on datasets outside the datasets they were originally chosen from. The top ten most cited violations and accidents were chosen because they were theorized to have the highest probability of success. To further analyze the quality of the predictions each prediction was broken back down into recall and precision. There were four possible results from this test being:

- Good: Greater than 50% recall and precision values.
- Okay: Greater than 50% recall value but less than 50% precision value.
- Bad: Greater than 50% precision value but less than 50% recall value.
- Terrible: Less than 50% recall and precision values.

## Results

Table 4 shows the summarized results of the 4,320 tests performed for the initial testing phase. This table illustrates the best hyperparameters to be used moving forward in the primary testing phase. Using AUC as the cutoff metric yielded a higher mean F1 (0.393) than using F1 directly (0.337), a difference that was statistically significant (*p* < 0.05). Likewise, GRU models (mean F1 0.393) outperformed LSTMs (0.375), and a learning rate of 0.001 was optimal, performing significantly better than 0.01 or 0.0001. Increasing the number of hidden layers degraded performance – a single-layer network was best (0.392 mean F1-Score), with performance dropping as layers increased.

**Table 4:** Summary of initial testing results F1-scores (MSHA 2025a, Minitab 2025)

Parameter	Option	Mean	Difference (*p*-value < 0.05)	Best option
Cutoff metric	AUC	0.393	Statistically significant	AUC
	F1-Score	0.337		
Model	GRU	0.393	Statistically significant	GRU
	LSTM	0.375		
Learning rate	0.01	0.346	Statistically significant between 0.001 and remaining options	0.001
	0.001	0.392		
	0.0001	0.343		
Number of hidden layers	1	0.392	All statistically significant	1
	2	0.337		
	3	0.300		
	4	0.273		
	5	0.223		

Table 5 presents the highest mean F1-score achieved for an accident prediction model and the parameters of that model, as well as the models (and parameters) that achieved highest recall and highest precision, respectively. The same is shown for violation predictions. Data processing proved to improve the quality of the algorithm due to each optimal F1-Score algorithm employing a data processing method. The violations were deemed more reliable to predict based on the best performing F1-score algorithm for violations having a higher mean F1-score than the algorithm for accidents. One reason for the stronger predictive performance on violations is the more regular occurrence of violations. The size of the accident and violation datasets was the same because they were broken up by time, but the violation dataset had many more natural occurrences as demonstrated back in Fig. 6 where the number of accidents and violations could not be displayed on the same axis due to their extreme variation. This resulted in the chosen data processing method generating less inferred points and thus a more representative dataset. Secondly, violations happen in a more systematic way (often tied to routine inspections and compliance issues), providing the model with more data points and clearer temporal patterns to learn. In contrast, accidents are relatively rarer and may be influenced by many random factors, making them inherently harder to predict with high accuracy.

**Table 5:** Results of primary testing phase: parameter settings for the best-performing models on the target accident (4th most frequent) and target violation (3rd most frequent)

Dataset	Metric	Mean	Number of points observed	Percentage of training dataset per iteration	Data processing method	Data processing division	Activation function
Accidents	F1-Score	0.479	15	2.00%	SMOTETomek	50/50	tanh
	Recall	0.858	5	6.67%	ADASYN	70/30	ReLU
	Precision	0.731	5	4.00%	none	60/40	tanh
Violations	F1-Score	0.740	5	1.00%	SMOTE	70/30	Leaky ReLU
	Recall	0.989	10	6.67%	SMOTE	70/30	ReLU
	Precision	0.881	15	4.00%	ADASYN	50/50	Leaky ReLU

Three models are identified for each dataset, one optimized for F1-score, one for recall, and one for precision (mean values shown)

From the general factorial regression with a 95% confidence level test, every interaction in both datasets were deemed significant except for the interaction between all variables simultaneously in the accident dataset (*p*-value of 0.898) and all variables simultaneously in the accident dataset minus the activation function variable (*p*-value of 0.675) (Minitab 2025). This confirms that each of the tested factors (sequence length, training data percentage, oversampling method, oversampling ratio, activation function) had a statistically significant impact on F1-score, underscoring the importance of tuning these parameters.

**Fig. 7:**
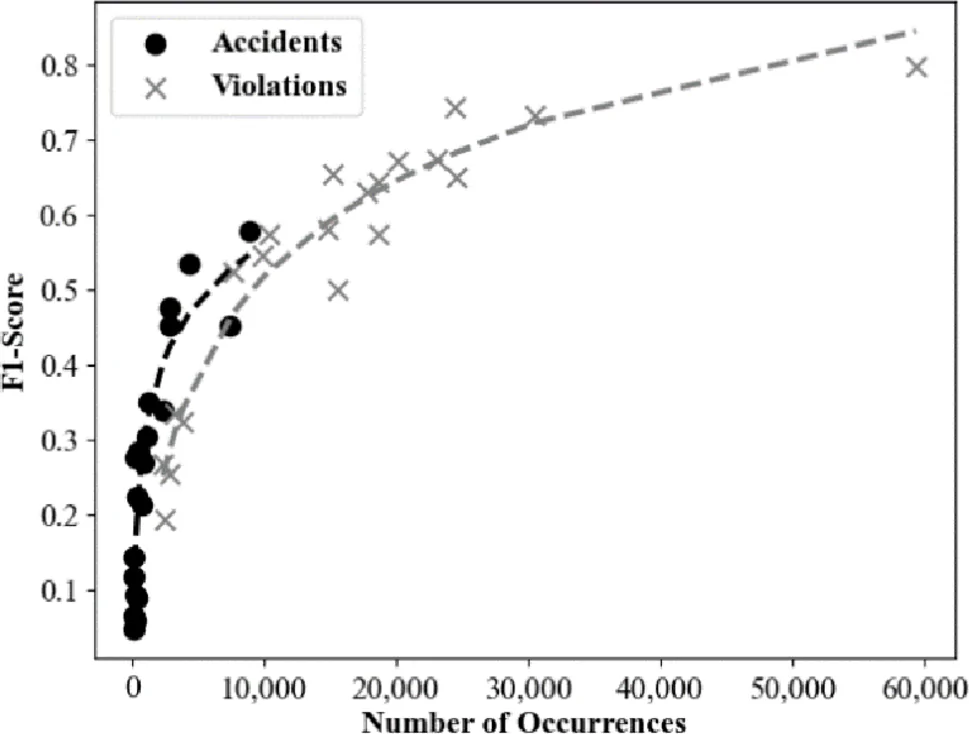
F1-Score versus number of occurrences for the 20 most cited accidents and violations in underground coal dataset (MSHA 2025a)

The results of phase one of the final testing can be observed in Fig. 7. The violation trendline has an *R*^2^ value of 0.878, and the accident trendline has an *R*^2^ value of 0.936, illustrating a strong relationship between the number of occurrences and the F1-score. The relationship between F1-score and number of occurrences also decreases as the number of occurrences increased in each dataset. Phase 1 of the final testing phase was the first instance of there being not enough positive points for training. When a given point had not occurred in sufficient quantity, an error would normally occur at the data processing stage due to the program attempting to add or subtract points based on an insufficient number of positive points. This failure point was when there were approximately ten positive entries in the dataset. This resulted in certain accidents and violations that were unable to be tested using the currently selected parameters. The average accident F1-Score is once again consistently lower than the violation F1-score as observed in the primary testing stage.

**Fig. 8:**
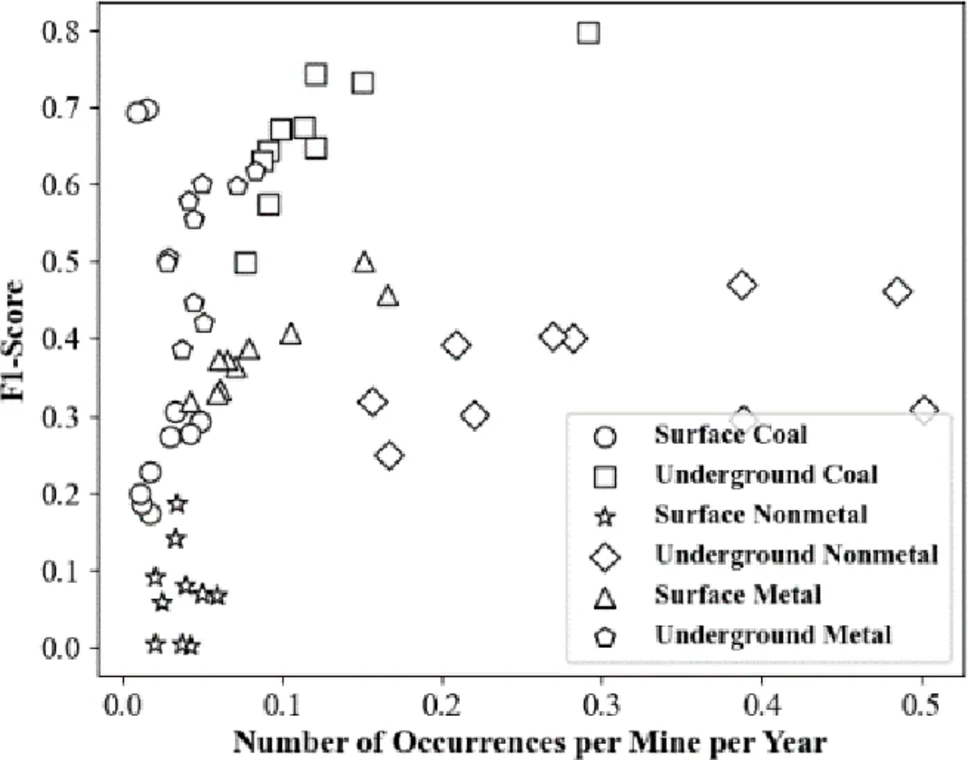
Violation F1-Score by mine type and mined commodity comparing F1-Score versus number of occurrences per mine per year (MSHA 2025a)

**Fig. 9:**
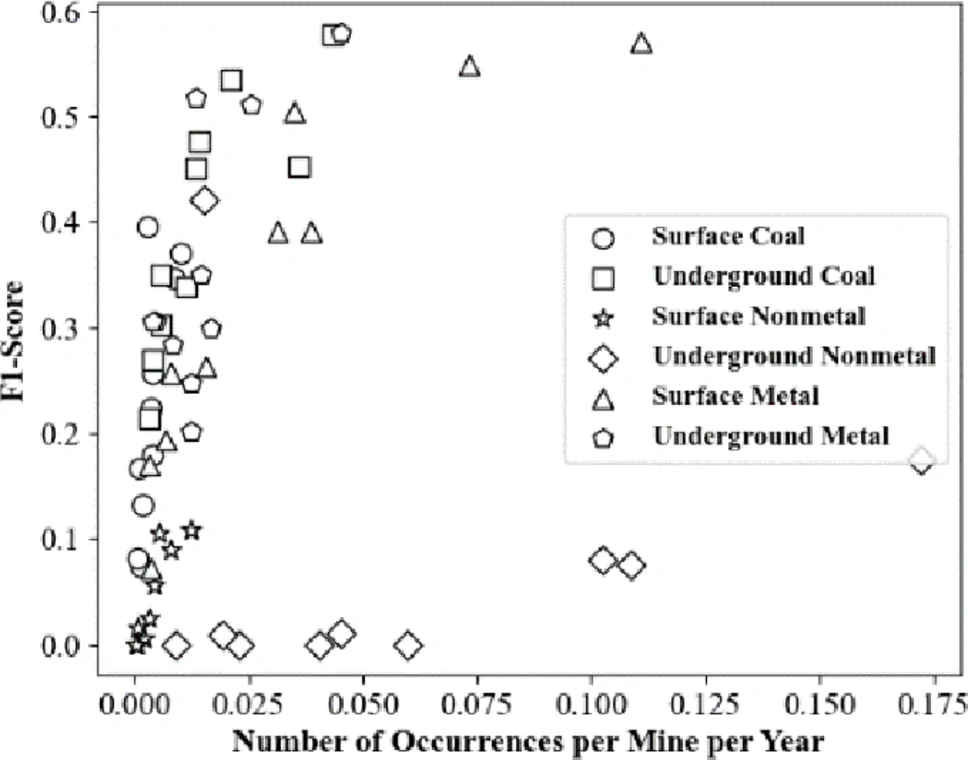
Accidents F1-Score by mine type and mined commodity comparing F1-Score versus number of occurrences per mine per year (MSHA 2025a)

Results from phase two of the final testing can be seen in Figs. 8 and 9. The trend observed in Fig. 7 with there being a positive but decreasing relationship between F1-Score and number of occurances is also observed in the data from phase two. The main outlier is the underground nonmetal datapoints which still follow the trend of F1-score increasing with number of occurrences but at a lower magnitude than the other datasets. The datapoints broken down into precision and recall can be viewed in Table 6. The highest performing dataset was the underground coal dataset followed by the surface and underground metal. The majority of points fell into the “terrible” category with 73% of the accident and 43% of the violation points.

**Table 6:** Results from phase 2 of final test where the highest F1-Score algorithm was used to predict the top ten most cited accidents and violations for both underground and surface mines for each commodity (MSHA 2025a)

Mine	Accidents				Violations			
	Good	Okay	Bad	Terrible	Good	Okay	Bad	Terrible
Underground coal	1	2	2	5	8	2	0	0
Surface coal	0	1	0	9	2	0	0	8
Underground nonmetal	0	0	2	8	0	0	4	6
Surface nonmetal	0	0	0	10	0	0	0	10
Underground metal	0	3	1	6	2	8	0	0
Surface METAL	1	2	1	6	0	10	0	0
Total	2	8	6	44	12	20	4	24
Percentage of Total	3%	13%	10%	73%	20%	33%	7%	40%

## Discussion

These models are intended to be used in tandem with a mining engineer and not as the sole alert system. In practice, this means the model would act as a decision-support tool. For example, a safety engineer could consult the model’s predictions for guidance on which types of incidents are at elevated risk in the next quarter, and then combine that insight with their expert judgment to form an action plan. The model is not meant to replace human decision-making but to augment it by drawing attention to patterns that might not be obvious from raw data.

**Fig. 10:**
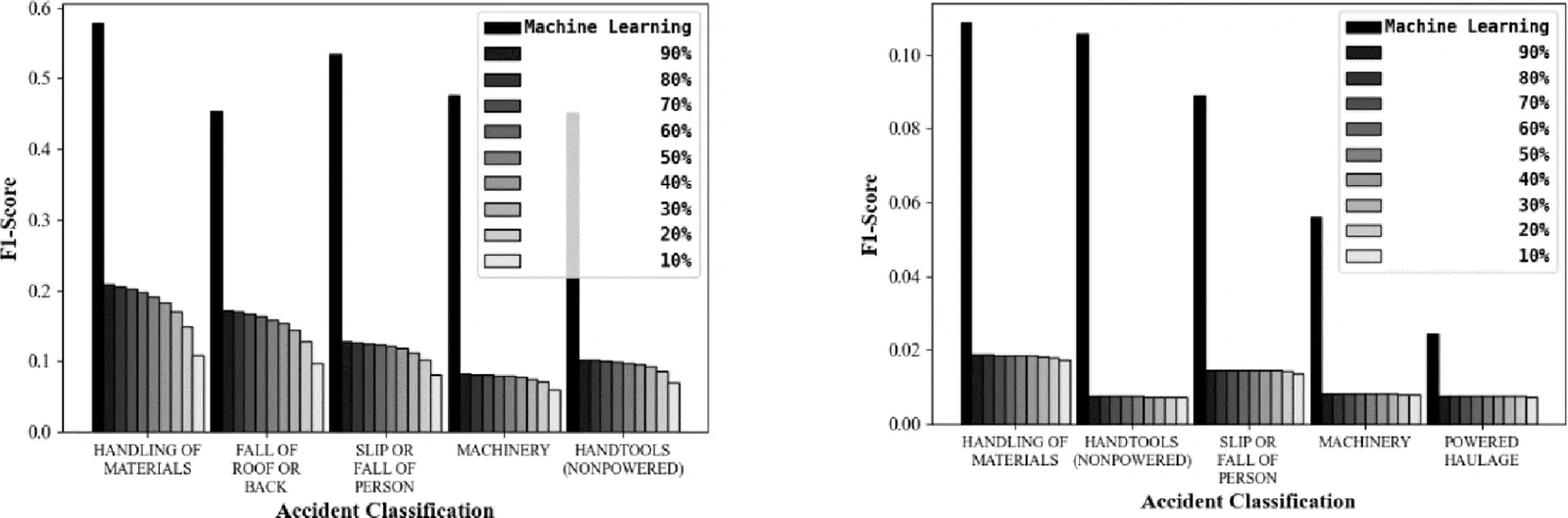
Machine learning algorithm results compared against random chance for top five accidents in ‘underground coal’ dataset (left) and ‘surface nonmetal’ dataset (right)

For this reason, the algorithms were compared to if the prediction was conducted by random chance. The calculations for these tests were based on the percentage of incidents the mining engineer would predict an accident would occur. Equations (16)-(19) outline how the results for the random chance were calculated based on the percentage of points the mining engineer predicted an event would occur (D) from the total number of positive points (P) and negative points (N) in the testing dataset for each dataset being tested. Percentages ranged from 90% to 10% and the F1-score for each was calculated. Figure 10 compares our model’s F1-scores to the F1 achieved by random guessing at various rates. Even the ‘terrible’ category models (low recall/precision) perform better than random chance predictions for the same scenarios. If the datasets were balanced, then a 50% acceptance rate would result in a 0.5 F1-score. These calculations highlight how imbalanced these datasets are.

16  P*D=TP

17  P*(1-D)=FN

18  N*(1-D)=TN

19  N*D=FP

### Improving the models using existing information

Though the algorithms outperformed random chances there are still opportunities for improvement. From the data already tested in this study, the best performing precision and recall algorithms can be used in tandem with the F1-Score algorithm to generate three predictive models. These algorithms have different priorities allowing for a risk assessment range to be developed. The precision algorithm prioritizes minimizing false positives, making its model the most reliable when it predicts incidents (most aggressive). The top performing recall algorithm prioritizes minimizing false negatives, allowing its model to have the greatest number of true positive points (most conservative). With this scale a mining engineer can be more informed in their decision making. To implement these algorithms phase one of the final testing stage was performed again but using each algorithm to examine if the trends remained constant.

**Fig. 11:**
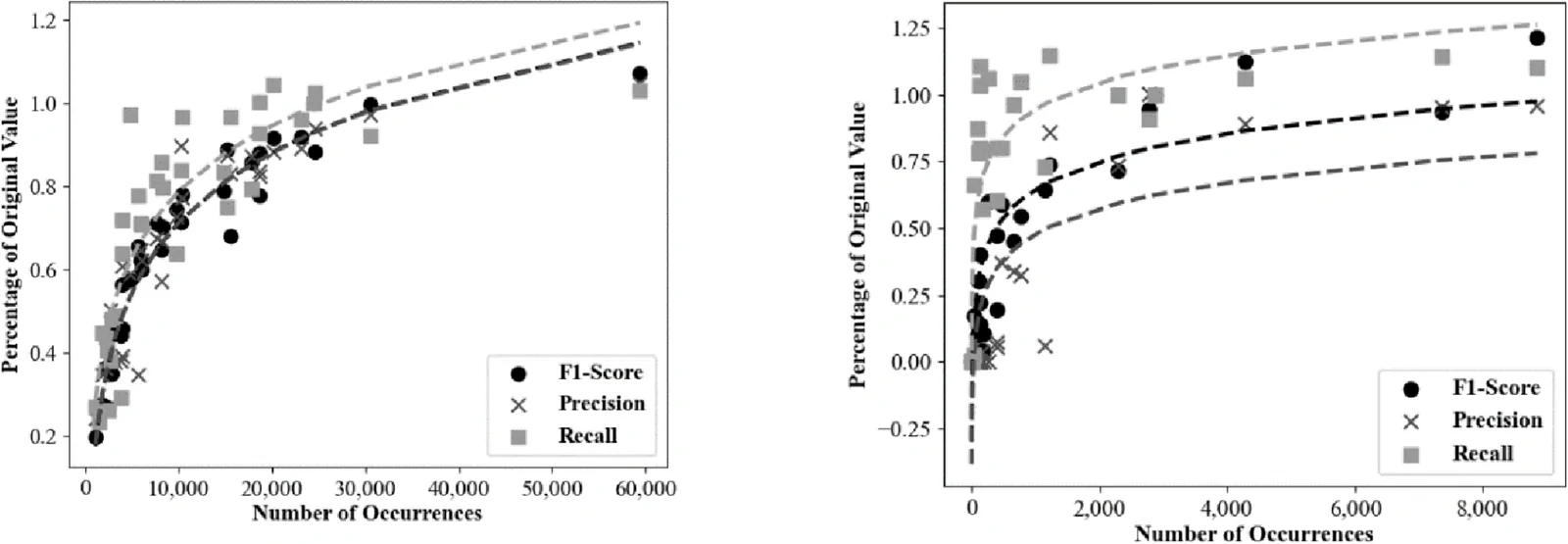
F1-Score, precision, and recall algorithms for 20 most cited accidents (right) and violations (left) in the underground coal dataset (MSHA 2025a)

Figure 11 shows these results for violations (left) and accidents (right). Instead of the *y*-axis being F1-score. In these figures, each model’s performance is normalized by its result on the base incident (the incident used to select that model – accident #4 for the accident models, or violation #3 for the violation models). This normalization allows us to compare the trend each model exhibits as incident frequency changes, despite their different absolute performance levels. The nearly parallel trends of the F1-score, precision, and recall-optimized models indicate that all three degrade in a similar way for low-frequency incidents and improve for high-frequency incidents. This consistency suggests we can confidently use the three models in parallel to provide a range of predictions (from conservative to aggressive) without one model unexpectedly outperforming the others on certain incidents.

**Fig. 12:**
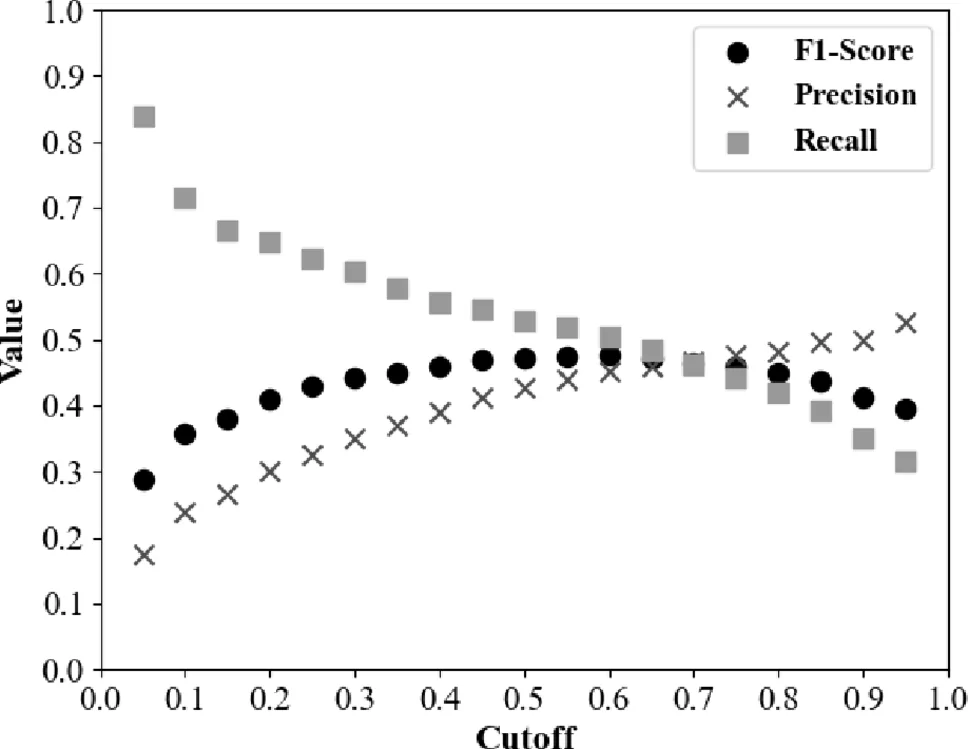
Changing cutoff metric for best performing F1-0Score algorithm to predict the forth most cited accident in the underground coal dataset (MSHA 2025a)

In fact, one can mimic the behavior of separate recall- or precision-optimized models by simply changing the classification threshold on the original F1-optimized model. Figure 12 shows the effect of varying the cutoff from 0.05 (very sensitive, high-recall) to 0.95 (high precision). The best recall (at cutoff 0.05) and best precision (at cutoff 0.95) from this approach were statistically compared to the actual best recall-optimized and precision-optimized models (Table 7). There was no significant difference in recall or F1 between using the 0.05 cutoff and training a separate recall-maximizing model (Minitab 2025). For precision, the separately trained model had higher precision than the 0.95-threshold model, but interestingly the 0.95-cutoff model achieved a higher F1-score (Minitab 2025). This suggests that adjusting thresholds on a single model can be an effective strategy to navigate the precision-recall trade-off, potentially obviating the need for maintaining multiple different models.

**Table 7:** Comparing best performing algorithms to best performing cutoffs from F1-Score algorithm when predicting fourth most cited accident in underground coal database based on a 95% confidence level (MSHA 2025a, Minitab 2025)

Item	Metric tested	F1-score
Recall	No difference	No difference
Precision	Algorithm	Cutoff

### Improving the models through additional testing

Further improvements to the models have been inferred based on trends observed in tests performed. The underground coal dataset performed the best when compared to the other datasets in phase two of the final testing. This could have been due to the underground coal dataset being used to identify the most optimal algorithm’s hyperparameters in the initial and primary testing stages. By rerunning the initial and primary testing stages with each individual dataset, the appropriate algorithm could be used for each dataset. This would mean that different trends in each dataset are better recognized by different hyperparameters. This was not investigated in this study due to time and computer processing limitations.

## Conclusions

This project investigated the use of a temporal machine learning algorithm to train a prediction model using the MSHA accident and violation datasets. Violations were categorized by *Part Section*, and accidents were categorized by accident classification. Violations proved more reliable to predict than accidents. However, we found that combining multiple models (each prioritizing a different performance metric) or simply varying the classification threshold allows us to develop a risk-level scale of predictions. This provides mine operators with richer information, not just whether an incident will occur, but an indication of the confidence or risk associated with that prediction. The model always performed best on the dataset from which it was originally selected, and worse on others, indicating a degree of overfitting to that dataset’s characteristics. In other words, model performance is highly data-dependent; using different data (e.g., another mine type) or suboptimal hyperparameters can lead to substantially different outcomes.

In summary, the contributions of this study are: (1) a novel application of temporal deep learning models (LSTM/GRU) to predict specific categories of mining accidents and violations, (2) an extensive evaluation of these models on different mine types and commodities, providing insight into the varying predictability of safety incidents, (3) introduction of a risk-based predictive framework that combines models/thresholds to balance false negatives and false positives, and (4) guidance on handling highly imbalanced, large-scale safety data for predictive analytics.

From these results new procedures can be developed to likely further improve results. Based on not just the hyperparameters themselves but the interactions between hyperparameters being deemed a factor in the results of the general factorial regression test in the primary testing stage, the initial and primary testing stages should be conducted concurrently. This means that each variation of hyperparameters tested in the initial should be tested with each variation of hyperparameters in the primary so each interaction can be experienced before optimal algorithms are selected.

Furthermore, the initial and primary testing should be rerun on different datasets. This is emphasized by the underground coal dataset performing the best in phase two of the final testing leading to the possibility that similar results could be exhibited by the remaining datasets if they were used to decide the most optimal algorithms.

These have not currently been tested due mainly to time limitations. The primary testing took thirty days of continuous runtime and based on the systems used in this study, combining the initial and primary testing phases would require more than five years of runtime. Performing the initial and primary tests for each dataset would have multiplied the time required for this study six times. These problems can be subverted with improved hardware or running multiple computers in parallel.

Ultimately, our findings highlight both the promise and the current limitations of temporal machine learning in mine safety. While the models perform better than random guessing—demonstrating that there is learnable signal in the incident history—the absolute performance for many incident categories remains modest. This underscores the challenge of predicting rare events and the need for continued research, whether through more advanced models, additional predictive features (e.g., operational or environmental variables), or more extensive training data. By addressing these challenges, future work can build on this study to move closer to deployable early-warning systems for mine safety.

## Data Availability

The datasets used and/or analyzed during the current study are available from the corresponding author on reasonable request.
